# Public Pharmacists' Session Ends

**Published:** 1915-05-08

**Authors:** 


### Public Pharmacists' Session Ends.

THE Public Pharmacists' and Dispensers' Association concluded its winter programme at the St. Bride Institute, E.C., on Wednesday, April 28. Mr. J. Hassall France (chairman of the Association) presided over a good gathering of representative institutional pharmacists, including Messrs. Broad, Heworth, Jenkin, Lindsey, Lindsay, Welford, Wolstenholme, Gibson (hon. secretary), and others.

In the absence, through bereavement, of Mr. Goodall (pharmacist at the London Skin Hospital) his paper on the "Life and Training of a Chemist's Apprentice Half a Century Ago" was read by the Secretary. It gave a graphic account of the varied nature of the duties performed by the embryo chemist in those days. Apprenticeship was for a period of seven years instead of the three or four years which now obtain. A copy of the legal indenture of a chemist's apprentice was incorporated in Mr. Goodall's paper, and its reading caused some amusement. The hours were long; no holidays other than Christmas Day, Good Friday, and half-days on Easter and Whit Mondays were given, and no annual leave! With the present dearth of pharmaceutical apprentices a reversion to the old-time rules would hardly increase the chemist's prospects of obtaining pupils.

Mr. H. C. T. Gardner, F.C.S., late chief pharmacist at the Miller Hospital, who is now in a responsible position with a large firm of chemical manufacturers in India, sent his experiences as a "Pharmacist in the Far East." He gave a vivid account of the European resident's life, followed by a detailed description of the various native customs, shrines, temples, etc. As to pharmacy, it seems there is no statutory qualification required to sell poisons, etc., as in this country, and armies of "drug-sellers" abound.

Mr. R. Welford (Colney Hatch Mental Hospital) described a very handy device for printing labels at a small cost for the dispensary bottles. The labels exhibited as specimens were both legible and neat, and would tend to "smarten up" the dispensary. Various systems of indexing the dispensary stock for the convenience of medical officers dispensing in emergency cases were also explained.

The question of approaching the Army Council concerning the status of pharmacists in the R.A.M.C. was considered, and this matter was left in the hands of the chairman and secretary.

Mr. Lindsey proposed a vote of thanks to the chairman for his able guidance during a trying period, and expressed the hope that next session would commence under happier conditions in the country.

